# Novel *Plasmodium falciparum Kelch13* polymorphisms in Cameroon with structural and physicochemical impact

**DOI:** 10.1128/aac.00884-24

**Published:** 2025-04-14

**Authors:** Loick P. Kojom Foko, Jahnvi Jakhan, Geetika Narang, Joseph Hawadak, Carole E. Eboumbou Moukoko, Vineeta Singh

**Affiliations:** 1 Parasite & Host Biology, ICMR-National Institute of Malaria Research 28861 , New Delhi, Delhi, India; 2 Academy of Scientific and Innovative Research (AcSIR) 550336 , Ghaziabad, Uttar Pradesh, India; 3 Department of Biological Sciences, Faculty of Medicine and Pharmaceutical Sciences, The University of Douala 469909 , Douala, Littoral, Cameroon; 4 Malaria Research Unit, Centre Pasteur Cameroon, Douala, Cameroon; 5 Laboratory of Parasitology, Mycology and Virology, Postgraduate Training Unit for Health Sciences, Postgraduate School for Pure and Applied Sciences, The University of Douala 231173 , Douala, Littoral, Cameroon; Columbia University Irving Medical Center, New York, New York, USA

**Keywords:** *Plasmodium falciparum*, *Kelch 13*, artemisinin partial resistance, polymorphism, *in silico *analysis, Cameroon

## Abstract

The recent emergence of *Plasmodium falciparum* (*Pf*) parasites resistant to artemisinin-based combination therapies (ACT) in Africa has outlined the need for continuous molecular surveillance of artemisinin partial resistance. Here, the genetic polymorphism in the *Kelch* 13 gene (*pfk13*) and its structural impact were analyzed. *Pf*DNA was extracted from dried blood spots of symptomatic and asymptomatic individuals living in different epidemiological facets of Cameroon. The *pfk13* gene was amplified by nested polymerase chain reaction, and amplicons were sequenced to detect single nucleotide polymorphisms (SNPs). The evolutionary history and the impact of the polymorphisms on physicochemical properties, structure, and function of the pfK13 protein were appraised using various *in silico* models. A total of ten SNPs were identified in this study, of which five non-synonymous SNPs have not been previously reported (L647**F**, D648**V**, N657**S**, K658**R**, and L663**P**). The genetic diversity of *pfk13* sequences was low, and the *pfk13* gene evolved under the neutral model. Some mutations, especially L663**P**, appeared to affect the function and structure of the pfK13 protein. Analysis of the physicochemical properties of the Cameroonian pfK13 protein sequences revealed slight changes in the solvent-accessible surface area, isoelectric point, and hydrophobicity. The results support the ongoing use of ACTs in the study areas, given the absence of validated SNPs associated with artemisinin partial resistance. Computational findings suggest a possible deleterious effect of some novel SNPs on the pfK13 structure and/or function.

## INTRODUCTION

*Plasmodium falciparum* (*Pf*) is the leading malaria pathogen, responsible for the bulk of morbidity and mortality worldwide (1). The parasite is dangerous given its ability to provoke severe clinical attacks, complications, and death, particularly in children under five years of age and pregnant women (1). In 2022, malaria is estimated to cause ~249 million cases and ~608,000 deaths, with the majority of these statistics occurring in sub-Saharan Africa (sSA), where *Pf* is the dominant species (1). Other human malaria species including *P. vivax*, *P. ovale curtisi, P. ovale wallikeri,* and *P. malariae* (but not *P. knowlesi* and *P. cynomolgi*) are found on the continent and can also elicit severe complications and death in a small number of patients (2, 3).

Malaria burden due to *Pf* has been steadily decreasing since the introduction of artemisinin-based combination therapies (ACTs) as first-line treatment for uncomplicated malaria in most endemic areas, due to high rates of *Pf* resistance to the older drugs chloroquine and sulfamides (1). Six ACTs are recommended by WHO for the first- and second-line treatment of malaria (1); artesunate +amodiaquine (AS + AQ) (2), artesunate + mefloquine (AS + MQ) (3), dihydroartemisinin +piperaquine (DHA + PPQ) (4), artemether + lumefantrine (AL) (5), artesunate + sulfadoxine-pyrimethamine (AS + SP), and recently (6) artesunate + pyronaridine (AS + PY) (1). Unfortunately, ACT effectiveness has been threatened by the emergence and spread of drug-resistant *Pf* populations in the Greater Mekong Subregion, South East Asia, where artemisinin partial resistance is now well established (4–6). Recent reports have also outlined the independent emergence of ACT-resistant *Pf* parasites in Africa, which bears the major global *Pf* burden (7, 8).

**FIG 1:**
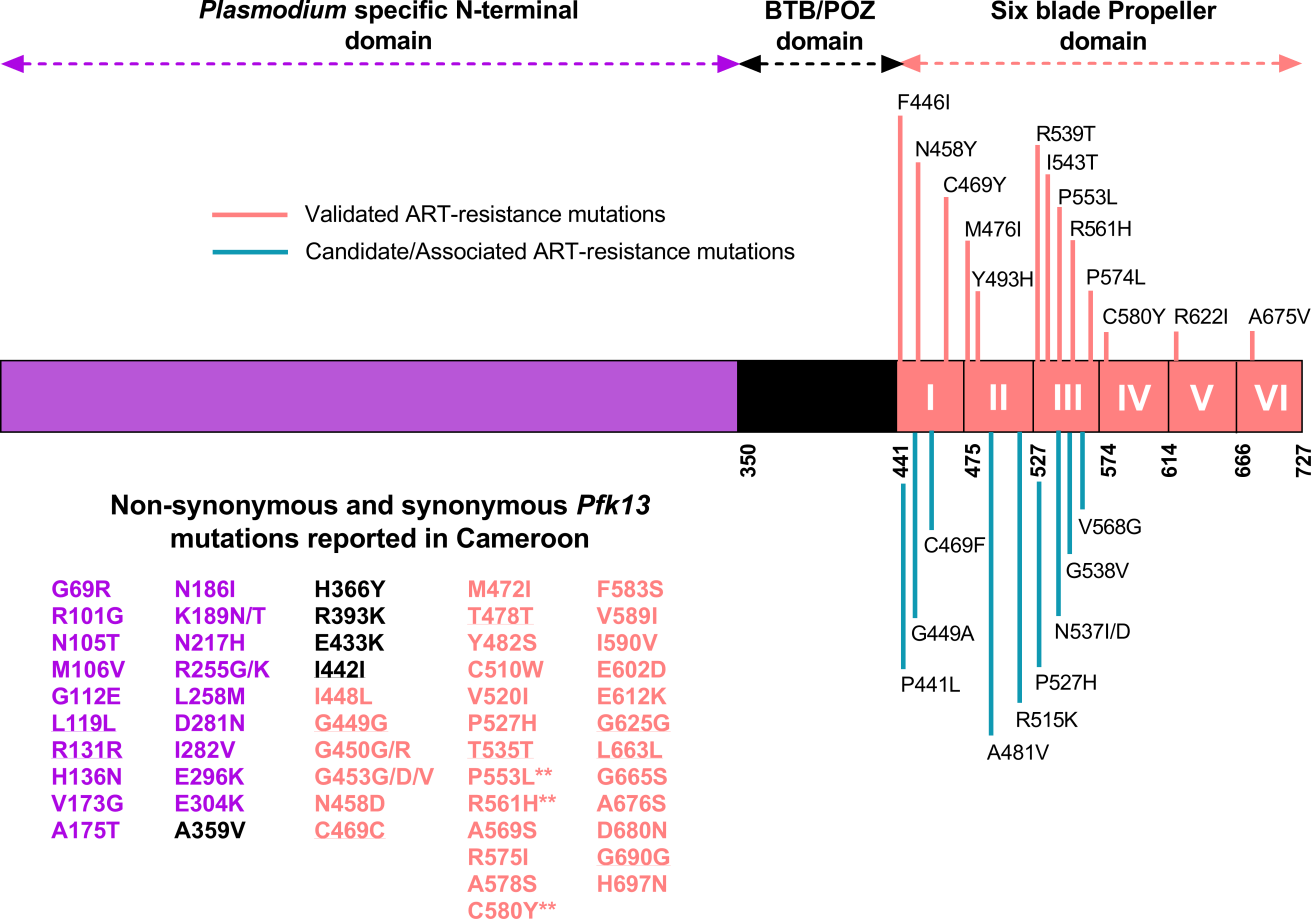
Schematic structure of the *pfk13* gene showing artemisinin-resistance validated and candidate/associated mutations, along with polymorphisms from autochthonous malaria cases reported in regions of Cameroon. Data on candidate and validated *pfk13* mutations associated with artemisinin resistance were retrieved from https://www.who.int/news-room/questions-and-answers/item/artemisinin-resistance (consulted 25/05/2024). The synonymous mutations are underlined. **Validated mutations associated with artemisinin partial resistance

Surveillance of antimalarial drug resistance is crucial for defining and implementing field-tailored drug policies and control measures. Molecular markers of drug resistance have appeared as the most widely used tool to track drug-resistant *Pf* parasites, compared to *in vivo* and *in vitro* studies, which are cumbersome and have poor standardization (9). Findings from genome-wide association and gene editing studies, coupled with evidence from *in vivo* studies, have identified non-synonymous mutations in the propeller domain of the *Kelch* gene (*pfk13*) as strong predictors of artemisinin partial resistance (10, 11). Of the >250 different *pfk13* mutations identified worldwide, thirteen (446I, 458Y, 469Y, 476I, 493H, 539T, 543T, 553L, 561H, 574L, 580Y, 622I, and 675V) are currently validated as markers of artemisinin partial resistance and clinical failure (Fig. 1) (12).

**FIG 2:**
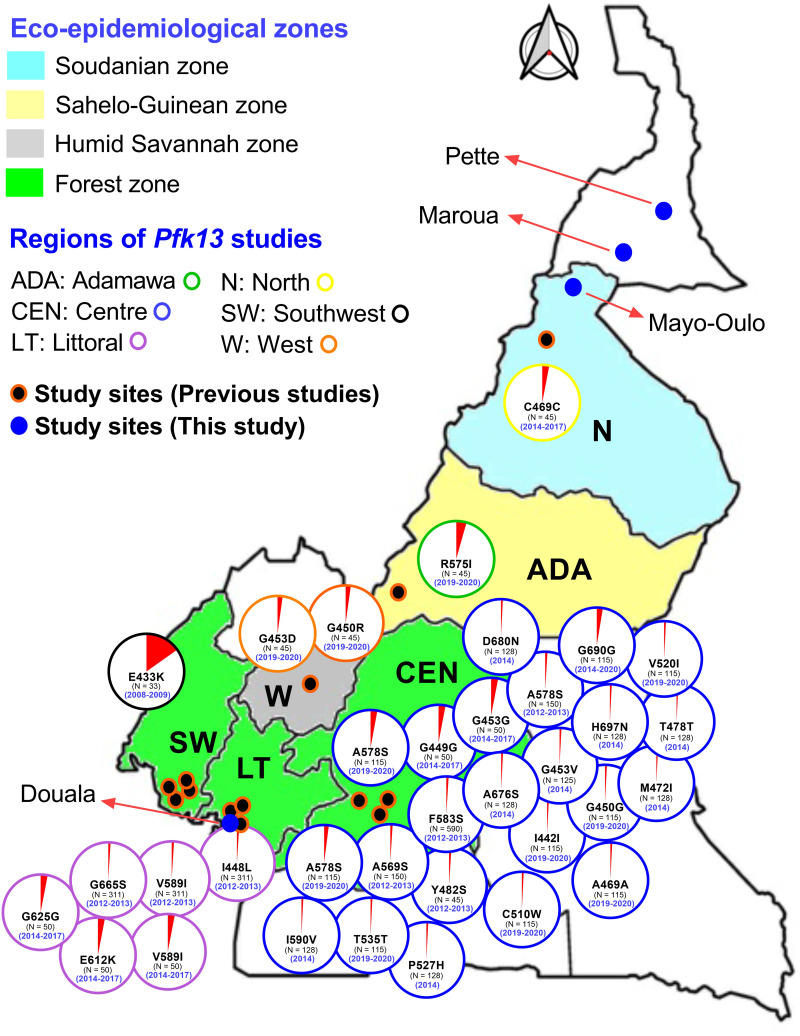
Overview of molecular epidemiological studies on mutations in the *pfk13* propeller domain of *Pf* parasites isolated from patients in Cameroon. Data used to generate the map were sourced from publications on indigenous malaria cases, peer-reviewed, published in English and French languages, retrieved from PubMed and Google, and not retracted were used to produce *pfk13* mutations and generate the map. The red slice of each pie chart depicts the proportion of each *pfk13* propeller domain mutation. The year of sample collection is presented in brackets. The maps were generated using the QGIS software v3.10 (https://qgis.org/en/site/)

Malaria due to *Pf* parasites is highly prevalent in Cameroon, where it is the first reason for medical consultations (~40 – 45%) and hospitalizations (~30 – 47%) (13–15). To achieve malaria control and elimination goals, the Government of Cameroon adopted AS +AQ as first-line treatment for uncomplicated malaria in 2004, followed by AL as an alternative in 2006 (13). Updated national treatment guidelines recommend three ACTs (AL, AS + AQ, DHA + PPQ) as the first-line treatment for uncomplicated malaria, while AS + PY is used as second-line treatment (13). To date, the effectiveness of ACTs remains high in sSA countries such as Cameroon (6, 16), but the recent independent emergence of artemisinin partial resistance in Rwanda, Uganda, Ethiopia, and Tanzania emphasizes the need for continuous surveillance of antimalarial drug resistance (7, 8, 17, 18). Existing data on molecular surveillance for *pfk13* mutations and artemisinin partial resistance are still limited in Cameroon (5, 19–32). However, several synonymous and non-synonymous polymorphisms in the *pfk13* non-propeller and propeller domains have been identified in *Pf* parasites collected from humans and mosquito vectors in Cameroon (Fig. 2) (5, 19, 25–29). Most of the *pfk13* genetic polymorphism data are available for five regions, especially the southwest and central regions, where a high diversity of mutations has been reported. In other regions, there are no data (e.g., East, South) or a lack of data (e.g., Littoral, North, and Far North) on the genetic diversity of the *pfk13* gene (Fig. 2 and https://doi.org/10.6084/m9.figshare.28124630). With this in mind, this study aimed to determine the genetic profile and evolutionary history of the *pfk13* gene of natural *Pf* populations from three epidemiologically distinct malaria regions of Cameroon.

## RESULTS

### Study samples and patient demography

**FIG 3:**
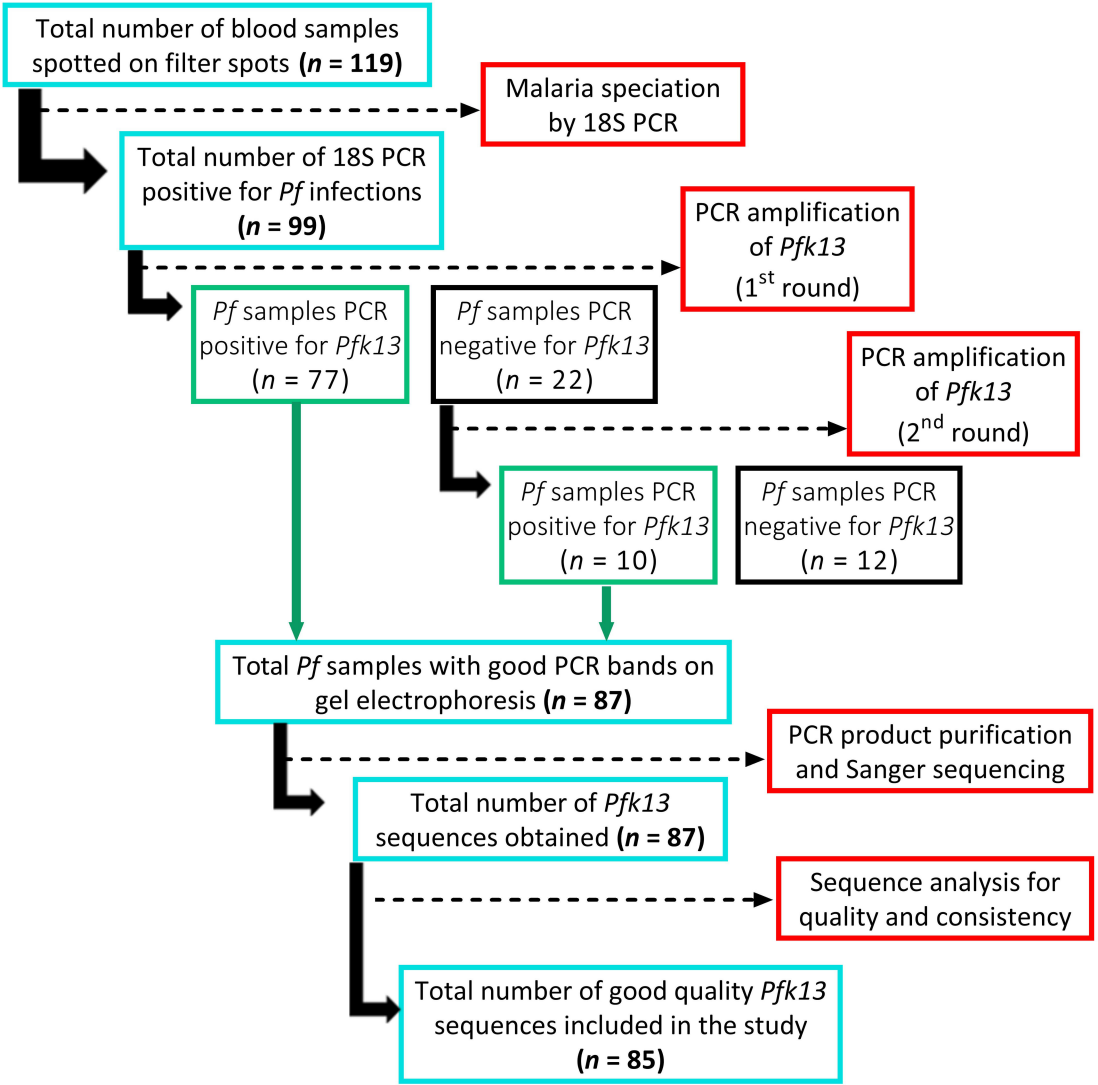
Flow diagram depicting the selection of *Pf* isolates included in the study. PCR: polymerase chain reaction; *Pf*: *Plasmodium falciparum.*

Of the 119 samples collected during the year 2019 (48 in Douala, 32 in Maroua, 13 in Mayo-Oulo, and 26 in Pette), 99 (83.2%) were PCR-positive for *Pf* infections, of which 77 samples were successfully amplified for the *pfk13* gene during the first round of amplification (Fig. 3). The remaining 22 samples were re-run, and 10 of them were successfully amplified in the second round of amplification. Based on gel electrophoresis analysis, 85 of the 87 successfully amplified samples yielded good quality PCR bands, and thus, were purified for sequencing. Finally, 85 good quality *pfk13* sequences (28 in Pette, 17 in Maroua, 7 in Mayo-Oulo, and 33 in Douala) were obtained and included in the study for analyzing genetic diversity and evolutionary patterns.

The sociodemographic and clinical characteristics of the patients, for whom good quality *pfk13* sequences were obtained, are summarized in Table 1. Males accounted for 54.5% of the patients in Douala, while females represented 60.7% in Pette, 76.5% in Maroua, and 85.7% in Mayo-Oulo. Patients from Douala were on average older than those from Pette (20.3 ± 15.9 years), Maroua (14.3 ± 11.4 years), and Mayo-Oulo (9 ± 9 years), but the difference was not statistically significant (*P* = 0.16). Few sequences were obtained from asymptomatic cases or submicroscopic parasitemia for which the highest rates were found in Maroua (29.4% and 41.2%, respectively) (Table 1).

**TABLE 1:** Details of patients with successful amplification of the *pffi13* gene^*a*^

	Forest	Sahelian		Soudanian		
Variables	Douala	Pette	Maroua	Mayo-Oulo	Total	*P*
Number of sequences	33	28	17	7	85	
Males, *n* (%)	18 (54.5%)	11 (39.3%)	4 (23.5%)	1 (14.3%)	34 (40%)	0.07^*b*^
Age ± SD	20.8 ± 16.6	20.3 ± 15.9	14.3 ± 11.4	9.0 ± 9.0	18.4 ± 15.2	0.16^*c*^
Age <5 years, *n* (%)	6 (18.8%)	2 (7.1%)	5 (29.3%)	3 (42.9%)	16 (19%)	0.13^*b*^
Asymptomatic parasitemia, *n* (%)	0 (0%)	0 (0%)	5 (29.4%)	1 (14.3%)	6 (7.1%)	0.0004^*b*^^,^^*d*^
Submicroscopic infection, *n* (%)	1 (3%)	0 (0%)	7 (41.2%)	0 (0%)	8 (9.4%)	< 0.0001^*b*,*d*^

^*a*^ Data are presented as frequency (percentage) and mean standard deviation (SD).

^*b*^ Pearson’s independence χ^2^ test was used to compare percentages.

^*c*^ Non-parametric Kruskal–Wallis test was used to compare mean values.

^*d*^ Statistically significant at *P* < 0.05.

### *Pfkelch13* propeller mutations

The results showed the occurrence of 10 SNPs, including three synonymous mutations (G449**G**, A617**A**, and L663**L**) and seven non-synonymous mutations (Q613**H**, L647**F**, D648**N**, D648**V**, N657**S**, K658**R**, and L663**P**), with different frequencies (Table 2). These *pfk13* gene mutations were detected in 10 out of the 85 sequences analyzed in this study. Interestingly, five of the seven non-synonymous SNPs (i.e., L647**F**, D648**V**, N657**S**, K658**R**, and L663**P**) had not been reported before the present study. The SNPs identified were mainly distributed in blade 5, while the two remaining SNPs were located in blade 1 (G449**G**) and blade 4 (Q613**H**). The status of the non-synonymous SNPs related to artemisinin partial resistance was unknown (Table 2). The majority of *pfk13* mutations were found in the town of Pette, a few mutations (i.e., G449**G**, A617**A**, and L647**F**) were reported in Douala, and no mutations were found in samples from Maroua and Mayo-Oulo.

**TABLE 2:** Profile of *pffi13*-propeller domain polymorphisms^*a*^

Nucleotide change	Amino acid change	Type of mutation	Validation status	Study sites (number of isolates)	Infection at the Codon analyzed^*c*^
1347 GGT **→**GG**G**	G449**G**	Synonymous	-^*d*^	Douala (*n* = 1)	Single
1839 CAA **→**CA**T**	Q613**H**	Non-synonymous	Unknown	Pette (*n* = 1)	Single
1851 GCC **→**GC**G**	A617**A**	Synonymous	-	Douala (*n* = 1)	Single
A1941**T** TTA **→**TT**T**	L647**F^*b*^**	Non-synonymous	Unknown	Pette (*n* = 6), Douala (*n* = 1)	Single
1942 GAT **→A**AT	D648**N**	Non-synonymous	Unknown	Pette (*n* = 1)	Single
1943 GAT **→**G**T**T	D648**V^*b*^**	Non-synonymous	Unknown	Pette (*n* = 1)	Single
1970 AAT **→**A**G**T	N657**S^*b*^**	Non-synonymous	Unknown	Pette (*n* = 1)	Single
1973 AAA **→CG**A	K658**R^*b*^**	Non-synonymous	Unknown	Pette (*n* = 1)	Single
1988/89 CTA **→**C**CG**	L663**P^*b*^**	Non-synonymous	Unknown	Pette (*n* = 1)	Single
1989 CTA **→**CT**T**	L663**L**	Synonymous	-	Pette (*n* = 1)	Single

^*a*^ All mutations have been cited under the domains of the *Kelch* gene region showing the codons with the mutation, the base and nucleotide changes, the type of mutation, the status of mutations as per WHO report, and the study site where they were observed. Bold and underlined = mutant allele; *n* = number of amino acid substitutions.

^*b*^ Novel mutations.

^*c*^ Single infection for the gene analyzed (i.e., *pffi13*) at a given codon was defined based on analysis of chromatogram peak. The presence of two concurrent peaks was considered as a mixture of wild-type and mutant at the given codon.

^*d*^ -, not applicable.

### Genetic diversity and evolution of *pfk13* sequences

The genetic diversity and evolution parameters for the *pfk13* gene responsible for artemisinin partial resistance are summarized in Table 3. A total of four haplotypes were found in all sequences, with the highest number of haplotypes found in Pette (three haplotypes). The haplotype diversity was low for the *pfk13* gene with values ranging from 0 in Maroua and Mayo-Oulo to 0.177 in Pette. The nucleotide diversities, measured independently by theta (θ) and Pi (π), were variable across the study areas, but the highest values for these diversities were found in Pette (π = 0.00025, θ = 0.00076). The test of neutrality as measured by TajD, Fu and Li’s D, and Fu and Li’s F tests were not statistically significantly deviated from the model of neutral expectation in any of the study areas (Table 3).

**TABLE 3:** Haplotype diversity of *pffi13* sequences in isolates^*a*^

	Forest	Sahelian		Soudanian	
Parameters	Douala	Pette	Maroua	Mayo-Oulo	Overall^*b*^
Number of sequences	33	22	17	7	79
Size of the nucleotide fragment	720	720	720	720	720
Number of haplotypes (H)	4	8	1	1	8
Haplotype diversity (Hd) ± SD	0.061	0.177	0	0	0.075
Number of segregating sites	3	6	0	0	8
Number of mutations	3	8	0	0	10
Number of single mutations	2	6	0	0	8
Total number of SNPs	1	2	0	0	3
Nucleotide diversity (π)	0.00008	0.00025	-^*d*^	-	0.00011
Nucleotide diversity (θ)	0.00034	0.00076	-	-	0.00084
TajD	−1.14014	−1.51481	-	-	−1.64103
Fu and Li’s *D*	1.71333	−2.10924	-	-	−3.28623^*c*^
Fu and Li’s *F*	−1.78961	−2.23805	-	-	−3.24598^*c*^

^*a*^ SD: Standard deviation, SNP: Single nucleotide polymorphism, Rm: minimum number of recombination events, Ra: estimated recombination between adjacent sites, Rb: estimated recombination per gene.

^*b*^ Six sequences from Pette were excluded from the analysis due to short length. Thus, 79 sequences were analyzed in this table.

^*c*^ Statistically significant at *P* < 0.05.

^*d*^ -, not applicable.

### Effect prediction of SNPs and physicochemical properties of the *pfk13* sequences

Some mutations found in this study (i.e., L647**F**, D648**V**, and L663**P**) and those validated (i.e., R539**T**, R561**H**, C580**Y**) appeared to impact the function and/or structure of the *Pf*K13 protein based on either SIFT, PolyPhen-2, or MutPred2. The L647**F** mutation, found in six isolates from Pette and one isolate from Douala, was predicted to be possibly damaging to PfK13 structure/function based on the PolyPhen-2 tool (Humdiv = 0.94). The D648**V** mutation found only in Pette (one isolate) was predicted to be deleterious (score = 0.596) based on MutPred2 results. Finally, the mutation L663**P** was predicted to be deleterious by both using the SIFT tool (score = 0) and the MutPred2 tool (score = 0.852). Finally, the majority of mutations induced changes in the ΔΔG using the DDGUN tool, with stabilizing effects (ΔΔG_(change)_ <0) for the Q613**H**, L647**F**, D648**N**, D648**V**, N657**S**, and K658**R** mutations; whereas the L663**P** mutation was predicted to induce destabilizing effects on the PfK13 protein with a ΔΔG_(change)_ of 1 (Table 4). Similarly, the validated mutations R539**T**, R561**H**, and C580**Y** were all predicted to be deleterious by the SIFT tool with respective scores of 0, 0.02, and 0.03 (Table 4).

**TABLE 4:** Effect prediction of the single nucleotide polymorphisms on structure and/or function and physicochemical characteristics of the pfK13 protein. The bold values outline signicant impact on PfK13 protein as per the different computational used (i.e., SIFT, MutPred2, Humdiv score)^*a*^

	Prediction analyses				Physico-chemical analyses				
SNPs	SIFT score^*d*^	MutPred2 score^*e*^	Humdiv score^*f*^	ΔΔG^*g*^	Number of residues	MW	SASA	IP	Hydrophobicity
Wild type	-^*h*^	-	-	-	726	83665	−0.681	5.67	0.727
R539**T^*b*^**	**0**	**0.608**	0	−0.4	726	83610	−0.676	5.61	0.728
R561**H^*b*^**	**0.02**	0.255	**0.99**	0.0	726	83646	−0.679	5.65	0.729
C580**Y^*b*^**	**0.03**	**0.766**	**0.99**	0.5	726	83725	−0.687	5.67	0.729
Q613**H**	**0.03**	0.302	0.003	0.2	726	83674	−0.681	5.72	0.726
L647**F^*c*^**	0.20	0.489	**0.94**	−0.1	726	83699	−0.683	5.67	0.725
D648**N**	0.09	0.261	0.002	−0.2	726	83664	−0.681	5.74	0.726
D648**V^*c*^**	0.10	**0.596**	0	−0.5	726	83649	−0.671	5.74	0.723
N657**S^*c*^**	0.16	0.146	0.54	0.1	726	83664	−0.671	5.67	0.724
K658**R^*c*^**	0.08	0.268	0	−0.4	726	83693	−0.682	5.67	0.723
L663**P^*c*^**	**0**	**0.852**	0.001	1	726	83648.9	−0.689	5.67	0.730

^*a*^ SNP: Single nucleotide polymorphism, PolyPhen-2: Polymorphism Phenotyping v2, SIFT: Sorting intolerant from tolerant, ΔΔG: Change in free energy before and after the mutations, MW: Molecular weight, IP: Isoelectric point, SASA: solvent accessible surface area.

^*b*^ These validated artemisinin resistance mutations were analyzed for comparative purposes.

^*c*^ Novel mutations.

^*d*^ SIFT score < 0.05, the non-synonymous change is predicted to be deleterious.

^*e*^ MutPred score > 0.500, the mutation is predicted to be deleterious to protein function/structure.

^*f*^ Humdiv score, the mutation is probably damaging (Humdiv ≥ 0.953), possibly damaging (0.953 > Humdiv ≥ 0.432), or benign (Humdiv ≥ 0.0024).

^*g*^ Non-synonymous mutation was classified as destabilizing (ΔΔG > 0), neutral (ΔΔG = 0) or stabilizing (ΔΔG < 0).

^*h*^ -, not applicable.

Analysis of physicochemical properties of Cameroonian *pfk13* sequences revealed slight changes in SASA, IP, and hydrophobicity compared to the wild type 3D7. The SASA values of the field sequences ranged between –0.689 and –0.671, while that of the wild type was –0.681. The IP of the field sequences was either similar to that of the wild type (IP = 5.67) for sequences carrying the mutations L647**F**, N657**S**, K658**R**, and L663**P**, or higher (IP range: 5.72–5.74) for sequences carrying the Q613**H**, D648**N**, and D648**V** mutations. Hydrophobicity values of field sequences were approximately lower than that of the wild type (hydrophobicity = 0.727), except for sequences carrying the L663**P** mutation (hydrophobicity = 0.730) (Table 4).

### Structural analysis of the *pfk13* mutations

**FIG 4:**
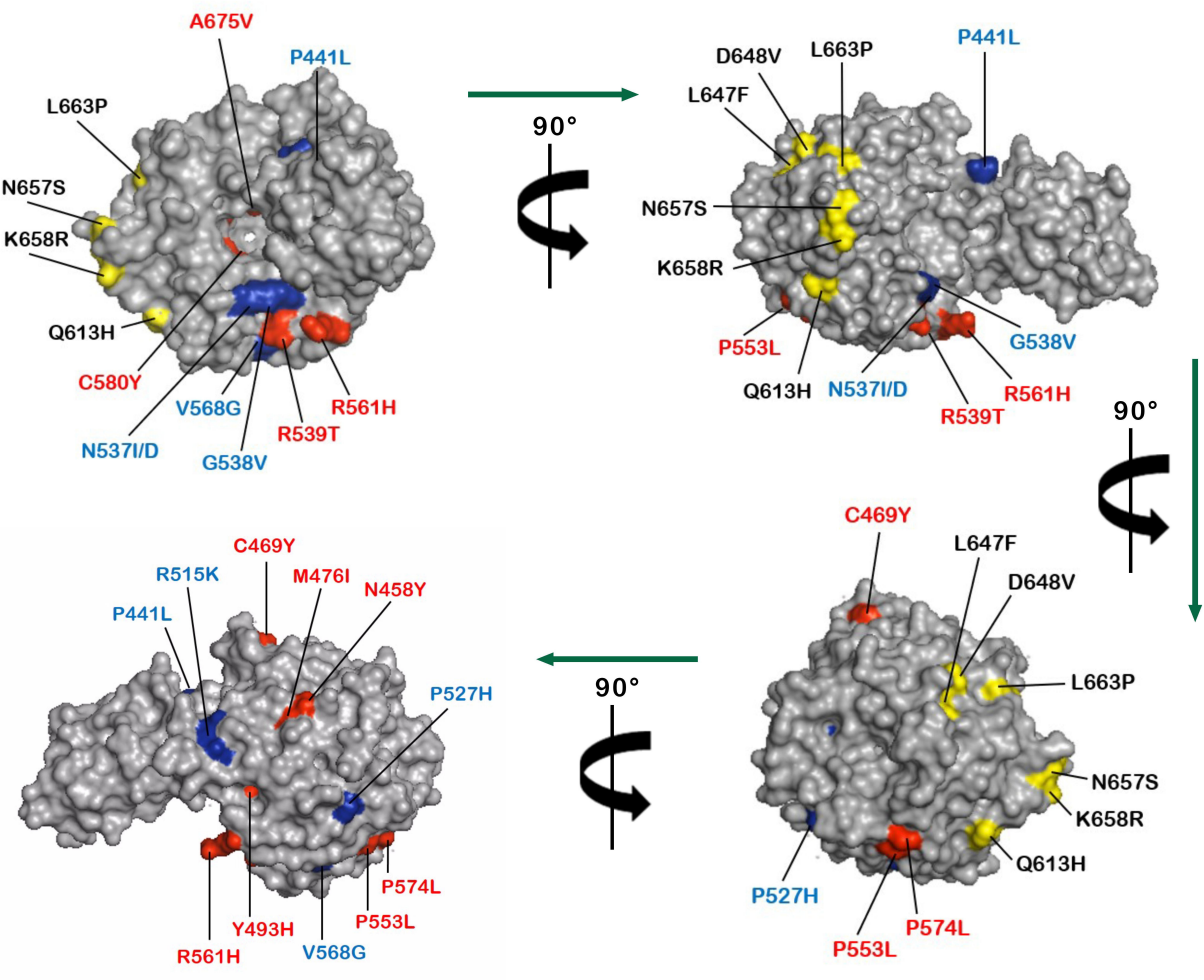
Molecular surface representation of the pfK13 protein showing orthogonal views of some non-synonymous SNPs found in the study. The mutations mapped included those validated with artemisinin-resistance (red), associated with artemisinin-resistance (blue), and novel SNPs found in the study (yellow). The PDB File of the *pfk13*-propeller domain (PDB ID 4YY8) was retrieved from The Research Collaboratory for Structural Bioinformatics Protein Data Bank (RSCB PDB).

The structural mapping of the non-synonymous SNPs found in the study, and the mutations validated or suspected to be associated with artemisinin partial resistance and ACT failure is presented in Fig. 4. The analysis seems to suggest that there is no concrete pattern in the spatial distribution of the mutations and their relationship with the resistance phenotype.

### Discussion

The emergence of ACT-resistant *Pf* parasites in the southern part of the African continent has alerted health and scientific communities and outlined the need to track and identify early signals of artemisinin partial resistance and adequately mitigate its spread in malaria endemic regions. In this study, we analyzed *pfk13* polymorphisms of *Pf* samples from different malaria transmission areas in Cameroon.

No *pfk13* mutations associated with artemisinin partial resistance were found in the study sites which is consistent with previous studies in Cameroon after 15 years of ACT implementation (Text S1). Similar results were found in recent studies conducted in India and South Sudan (33, 34). Other studies reported validated ACT resistance mutations (i.e., C580**Y** and R561**H**) or candidate artemisinin partial resistance mutations (i.e., P441**L**), at very low rates in Ghana, and the Democratic Republic of Congo (35, 36), whereas studies reported high rates (8%) of the candidate artemisinin partial resistance kelch13 622**I** mutation in Ethiopia (17). These findings are interesting as they suggest that artemisinin partial resistance has not yet emerged in Cameroon, and thus, support the ongoing use of ACTs in these areas. However, it is crucial to replicate such studies in other regions of Cameroon, where there is a dearth of molecular epidemiology studies on artemisinin partial resistance.

Several synonymous and non-synonymous SNPs were found in this study. The D648**N** mutation was found in one isolate from Pette, and was also found previously by Yang et al. (37) in migrant workers returning to Henan province, China from Ghana (37), and by Dafalla et al. (38) in Saudi Arabia (38). The synonymous G449G mutation was found in one isolate from Douala, and our previous work reported this mutation in the town of Yaoundé, Centre region (26). Finally, the Q613**H** mutation, found in one isolate from Pette, has been reported in Nigeria, Senegal, and China (39–41). The presence of these mutations in these areas could be due to either *de novo* emergence or gene exchange via human movements. A cocktail of host, parasite, and environment factors may modulate the emergence of such genetic polymorphisms. For example, drug pressure from self-medication is an important cause of the emergence of drug-resistant parasites. Several studies have shown that self-medication with antimalarial drugs is a common practice among the Cameroonian population (42–44).

We report here five novel non-synonymous polymorphisms (L647**F**, D648**V**, N657**S**, K658**R**, and L663**P**). All of these mutations were found in Pette, a town in the Far North region of Cameroon, where malaria transmission is high with epidemic peaks. One of these novel SNPs (L647**F**) was also reported in Douala, the economic country’s capital. It is unknown whether these mutations arose *de novo* or were introduced by human migration. In general, such polymorphisms are reported at marginal frequencies as found in the present study. This is likely due to the low nucleotide diversity in the *pfk13* gene reported in this study and others (45–47). The role of these mutations in ACT resistance is unknown, but they are unlikely to play a role in the acquisition of an artemisinin partial resistance phenotype. This assumption is supported by our computational findings, which indicated that these mutations do not congregate to any specific surface of the protein, suggesting that there is no clear relationship between the spatial distribution of these novel, validated, or candidate SNPs and their association with artemisinin partial resistance. Moreover, given the fact that few studies have reported (i) a persistence of *Pf* parasitemia on day 3 in ACT-treated Senegalese patients with wild-type *pfk13* alleles (48, 49), and (ii) statistically significant associations between late parasitological failure and three non-synonymous *pfk13* mutations (V692**G**, N664**I**, Q661**H**) in Nigerian patients (39). It should be interesting to confirm our hypotheses by *in vitro* or *in vivo* evaluation of the clinical significance of these mutations (50, 51).

Some mutations, including L663**P**, appeared to affect the function and structure of the pfK13 protein. Mohon and colleagues found that the A578**S** mutation was predicted to be deleterious to the structure/function of the pfK13 protein (52). Using molecular dynamics simulations, Copée and coworkers found that the pfK13 R539**T** and C580**Y** artemisinin partial resistance mutations were associated with some local structural destabilization of the Kelch-repeat propeller domain, but not in its shallow pocket (53). More recently, Yan and coworkers reported some structural alterations of the pfK13 protein, such as the loss of hydrogen bonds and conformational changes, associated with the F446**I** and C580**Y** mutations (54). Goel and colleagues also showed some subtle conformational changes of the pfK13 protein associated with the artemisinin resistance R539**T** and C580**Y** mutations, while no noticeable differences were found between the mutant A578**S** and wild-type protein (55).

The evolutionary analysis revealed a low genetic diversity of the *pfk13* gene, which is consistent with previous reports from different malaria endemic settings (45, 56–58). The mutations observed in this study could be due to normal genetic events (e.g., genetic recombination) or reflect a parasite adaptation to the drug pressure of antimalarial drug use. The TajD and Fu & Li’s values did not show any evidence of the effect of natural selection on the evolution of the *pfk13* gene. Consistent with previous studies in Kenya, Ethiopia, and India, this result suggests that the *pfk13* gene is evolving under the neutral model of molecular evolution in the study sites (57–61). In contrast, few authors reported high haplotype diversity, and the role of negative or purifying selection in the evolutionary dynamics of the *pfk13* gene in the West African region (56), Ethiopia (60), Kenya (60), and Ghana (47). Finally, Tandoh and colleagues found a role of balancing selection in the evolution of the *pfk13* gene in Ghana (62).

The results of the present study should be interpreted in light of its limitations. First, the small sample size of *Pf* sequences analyzed limits the generalizability of the findings at both regional and national levels. However, the study reports novel *pfk13* mutations and the absence of artemisinin partial resistance-associated mutations in the study areas, emphasizing the need for continuous artemisinin partial resistance surveillance in the country. Second, sequences were obtained using Sanger sequencing, which limited our ability to analyze and compare the genetic diversity of samples, especially those for which *pfk13* mutations were found. This approach, unlike more sensitive techniques such as whole-genome sequencing, limits the analysis and interpretation of data. Also, we were not able to perform deeper genetic relatedness analysis for samples from different areas carrying the same mutations (e.g., samples from Douala and Pette carrying the L647**F** mutation). Such findings would have further implications on the extent of the spread of specific K13 mutations in Cameroon. Finally, the analysis of the impact of novel mutations was performed only *in silico*. Therefore, it is crucial to conduct *in vitro* studies using gene-editing techniques to evaluate the role of the novel mutations in artemisinin partial resistance.

In conclusion, polymorphism in the *pfk13* gene has been analyzed in natural *Pf* populations from Douala, Pette, Maroua, and Mayo-Oulo. This study confirms the absence of circulation of ACT-resistant *Pf* parasites in the study areas. Few non-synonymous SNPs were found in the study, including five non-synonymous SNPs not previously reported. The Cameroonian sequences are genetically stable, are not under any evolutionary forces, and computational results suggest a possible deleterious effect of some non-synonymous SNPs (e.g., L663**P**) on the structure and/or function of the pfK13 protein. Continuing molecular surveillance and evaluating new polymorphisms *in vitro* and *in vivo* are key factors to continuing ACT use and preventing artemisinin partial resistance in Cameroon.

## MATERIALS AND METHODS

### Sample demographics

A hospital-based prospective cross-sectional study was conducted between April and August 2019 in four cities in Cameroon: Pette, Maroua, Mayo-Oulo, and Douala. All human malaria species, except *Pk*, have been documented in Cameroon, but *Pf* remains the predominant species (63–65). These towns are located in different regions of the country belonging to different epidemiological facets of malaria. Pette and Maroua are located in the Far North region and belong to the Sahelian epidemiological facet, which is characterized by a hyperendemic and seasonal malaria transmission, an entomological inoculation rate (EIR) of 10 infective bites/man/month, with epidemic outbreaks lasting 2–3 months during the rainy season. Mayo-Oulo (North region) belongs to the Soudanian epidemiological facet, as previously specified for the Sahelian facet, malaria transmission is hyperendemic and seasonal, with an EIR of ~10 infective bites/man/month. Douala (Littoral region) is the economic capital of Cameroon and is located in a forest area where EIR is ~100 infective bites/man/month, with holoendemic and perennial malaria transmission (65, 66).

We included all patients who meet the following criteria: i) permanent residents (at least 2 years) in the study area, ii) with no travel history in the past three weeks, and iii) with or without fever. All patients who met the inclusion criteria and volunteered to participate were enrolled. A survey form was used to collect patient data including demographics (gender, age), clinical presentation (presence of fever), and parasitological data (microscopy test result, parasitemia). Blood samples were collected by venipuncture, and then, blood drops were spotted on the Whatman filter (GE Healthcare Ltd., Amersham, UK) and air-dried for 15 minutes before transfer to the laboratory for further molecular investigation of *pfk13*–propeller domain polymorphism.

### DNA extraction and molecular detection of *Pf* infections

The dried blood spots were punched for plasmodial genome extraction according to the Qiagen protocol (QIAGEN blood DNA extraction kit, Valencia, California, USA). The extracted DNA was eluted in 100 µL of elution buffer (10 mM Tris–HCl; 0.5 mM EDTA; pH 9.0), and stored at −20°C until further molecular analyses. A nested polymerase chain reaction (PCR) based protocol developed by Snounou and colleagues was used for the specific detection of *Pf* infections by amplification of the 18S small subunit ribosomal RNA gene (67). Primers and thermal conditions are published earlier (67). Each amplification was performed in a total volume of 25 µL PCR reaction containing 12.5 µL of GoTaq Green Master Mix (Promega, USA), 1 µL of each primer (10 µM), 1 µL DNA template and free-nuclease water *quantum satis*. Negative and positive controls were performed in each experiment. Amplicons were loaded onto 2% agarose gel pre-stained with ethidium bromide for gel electrophoresis. Electrophoretic migration of amplicons was performed at 72 V for 1 h. Amplicons were visualized using an ultraviolet transilluminator. *Pf* infection was confirmed by the presence of a 205 bp amplicon on the electrophoretic gel. Only *Pf-*PCR positive samples were further used for molecular amplification of the *pfk13* gene (https://doi.org/10.6084/m9.figshare.28124630).

### Analyzing the *pfk13*-propeller domain gene

#### Sequencing

A nested PCR amplification method of the *pfk13*-propeller domain codons (> 440^th^ – 727^th^) codon was used following the protocol proposed by Ariey et al. (10). Standard primer sequences for the *pfk13*-propeller domain PCR amplification and cycling conditions were used (10). The composition of the master mix and electrophoresis gel conditions were similar to those used for PCR of the *18S* gene. Successful amplification of the *pfk13*-propeller domain is indicated by the presence of an 849 bp amplicon on the electrophoretic gel. The amplicons were then purified using a DNA GeneJET PCR purification kit (Thermo Fisher Scientific, Lithuania), and sequenced in forward and reverse directions using an in-house 3730XL DNA genetic analyzer (Applied Biosystems).

#### Identification of SNPs

The chromatograms of each *pfk13* sequence were viewed using Finch^TV^ software, and manual trimming and base calling were performed as needed for some sequences, and with the help of the online software Poly Peak Parser (68). This server was used to identify double nucleotide peaks indicative of multiple SNPs and to resolve problems such as software-induced erroneous nucleotide calls. Incorrectly inserted nucleotides that appear to be oddly spaced relative to neighboring nucleotides or that were omitted from the sequence. Setting parameters included 100 nucleotides per row, trimming of ≥50 nucleotides from both the 3’- and 5’-ends of the sequences, and a signal ratio cut-off of 0.50 (calculated as peak signal/max signal for each position). Signals above this ratio are called alternative bases (68). Sequences of poor quality (e.g., no clear distinction between nucleotide signals due to baseline noise, high frequency of ambiguous signals or peaks to peaks, irregularly shaped peaks, loss of resolution, or very short sequences) were excluded, and sequencing was repeated a second time whenever possible. This strategy of sequence analysis ensured the removal of poor-quality sequences, thus providing reliable and accurate data. Consensus sequences were generated by first aligning the forward and reverse reads for individual samples using BioEdit software. A consensus was generated from the resulting alignment by selecting the highest quality base and also performing manual base calling for regions of ambiguity.

To detect polymorphisms in sequences encoding the *pfk13* gene, we obtained the reference nucleotide sequence of the *pfk13* gene from the PlasmoDB database (accession number PF3D7_1343700). Reference and field sequences were aligned by the multiple sequence comparison by log-expectation algorithm (MUSCLE) using MEGA X software (69, 70). Both synonymous and non-synonymous SNPs were analyzed for each sequence. The proportion of each polymorphism was expressed as a percentage calculated by dividing the number of isolates containing a given polymorphism by the total number of isolates analyzed. All *pfk13* sequences were independently analyzed by authors LPKF and JJ to ensure that all the mutants were true mutants. External quality control was performed by senior authors JH and CEEM. Any discrepancies were resolved by discussion with supervising authors CEEM and VS. The generated *pfk13*-propeller domain sequences have been deposited in the GenBank database via Banklt under accession numbers PP478669–PP478753.

#### Genetic diversity

Several genetic diversity parameters were calculated using the DNA Sequence polymorphism computer program v6 (DnaSP6) (71), including the number of haplotypes (H), haplotype diversity (Hd), number of segregating sites, number of SNPs, total number of SNPs, and nucleotide diversity estimates (π and θ). Analysis of these parameters was performed for each study site and for the total number of sequences analyzed.

#### Neutral evolutionary genetic analyses

The *pfk13* DNA sequences were aligned using MEGA X software (69); and population genetic parameters were calculated for each study site. The neutral theory of molecular evolution was tested for the *pfk13* sequences by computing Tajima’s D (TajD), Fu & Li’s D, Fu & Li’s F, and recombination tests. The normalized difference between nucleotide diversity estimates (π and θ) is estimated by TajD, while differences between segregating sites in internal and external phylogenetic branches are estimated by Fu & Li’s D and Fu & Li’s F tests (72, 73). Negative values of TajD indicate the role of evolutionary forces such as selective sweep or bottleneck, whereas a positive value shows the effect of population contraction or balancing selection on the evolution of genetic sequences. These neutral evolutionary tests are used to identify the demographic and natural selection forces driving the evolutionary dynamics of genes. All the parameters were calculated using the DnaSP6 software (71).

#### Computational analysis of the impact of amino acid changes

The pathogenicity of novel non-synonymous *pfk13* SNPs in the structure and/or function of the pfK13 protein was analyzed using three machine learning-based online servers namely Polymorphism Phenotyping v2 (PolyPhen-2), MutPred2, and Sorting Intolerant From Tolerant (SIFT) (74–76). The Humdiv model was used to analyze mutations using PolyPhen-2, and non-synonymous change was classified as probably damaging (Humdiv ≥0.953), possibly damaging (0.953 > Humdiv ≥ .432) or benign (Humdiv ≥0.0024). A mutation with a SIFT score <0.05 or a MutPred2 score >0.50 was considered deleterious to structure/function. The delta-delta Gibbs energy value (ΔΔG_(change)_) or free energy change between wild-type and SNP variants was computed using DDGUN (https://folding.biofold.org/ddgun/) (77), based on the formula ΔΔG_(change)_ (kcal mol^−1^) = ΔG_(mutation)_ - ΔG_(wilt type)_. Mutations were classified as destabilizing (ΔΔG_(change)_ >0), neutral (ΔΔG_(change)_ =0), or stabilizing (ΔΔG_(change)_ <0).

The impact of non-synonymous SNPs on the physicochemical parameters, including hydrophobicity, isoelectric point, solvent accessible surface area (SASA) and thermal stability of the pfK13 protein was also determined. SASA was calculated using GetArea (http://curie.utmb.edu/getarea.html), thermal stability was calculated using Scoop (http://babylone.ulb.ac.be), and hydrophobicity was calculated using SoDoPe (https://tisigner.com/sodope). The 3D model of *pfk13* SNPs was visualized and mapped using PyMol software. The 3D structure of pfK13 from the reference strain 3D7 was retrieved from the Research Collaboratory for Structural Bioinformatics Protein Data Bank (RSCB PDB) (https://www.rcsb.org/structure/4yy8), and the PDB file was used to perform the above-mentioned computational analyses. The computational analyses were also performed for wild type and validated artemisinin resistance mutations R539**T**, R561**H**, and C580**Y** for comparative purposes. These three mutations were selected due to their recent emergence in Africa (i.e., R539**T** and R561**H**) and their high proportion in Asia (i.e., R539**T**, R561**H**, and C580**Y**) (5, 78, 79).

### Operational definitions

- **Fever** = Rectal temperature ≥38.0°C for children ≤ 5 years and axillary temperature ≥37.5°C for children > 5 years, teenagers, and adults (80).
- **Asymptomatic parasitemia** = Presence of *Pf* parasites without any clinical symptoms (80).
- **Symptomatic parasitemia** = Presence of *Pf* parasites with a clinical symptom (e.g., fever) (80).
- **Submicroscopic infections** = Infections in which malaria parasites were not detected by LM but detected by PCR (65). In general, submicroscopic infections have parasitemia below 100 parasites/µL (81).
- **Single infection =** Presence of a single chromatogram peak at a given codon. In contrast, the presence of two peaks (similar height or one of the peaks reached 50% of the height of the other peak) at a given codon was considered a mixture of wild type and mutant type (82, 83).

### Statistical analyses

Data of interest were keyed in an Excel spreadsheet (Microsoft Office, USA), coded, checked for consistency, completeness, and missing data; and then exported to the StatView v5.0 for Windows (SAS Institute, Inc., Chicago, USA) and GraphPad v8.01 for Windows (GraphPad PRISM, IBM, California, USA) for statistical analysis. Categorical variables were summarized as percentages and 95% confidence intervals (95% CI), while numerical variables were presented as mean ± standard deviation (SD) in tables and/or graphs as appropriate. Quantitative variables were tested for Gaussian distribution using the Agostino-Pearson test (84). Non-parametric tests were used to analyze variables that did not follow a Gaussian distribution. The 95% CIs were calculated as previously proposed (85). Pearson’s χ^2^ test for independence and Fisher’s exact test were used to compare proportions with respect to Cochran’s rule (86). The Kruskal–Wallis test was used to perform multiple nonparametric comparisons of means. A two-tailed *p*-value < 0.05 was considered statistically significant for all analyses.

## Data Availability

The original contributions presented here are included in the article/Miscellaneous File Not for Publication. Sequences generated are deposited to the NCBI nucleotide database under accession numbers PP478669–PP478753. The *pfk13* chromatograms have been deposited at the public database OSF under the address https://osf.io, and can also be requested from The ICMR-National Institute of Malaria Research (Dwarka, New Delhi, India, website: https://hindi.nimr.org.in, email: director-nimr@icmr.gov.in). Finally, further complementary data to the manuscript are freely available at https://doi.org/10.6084/m9.figshare.28124630. Further inquiries can be directed to the corresponding author. Benefits from this research accrue from the sharing of our data and results on public databases as described above.
